# Genomic Profiles of a Patient of Pulmonary Hepatoid Adenocarcinoma With High AFP Level: A Case Report

**DOI:** 10.3389/fonc.2019.01360

**Published:** 2019-12-11

**Authors:** Jinglin Li, Huiwei Qi, Bingxin Xu, Jing Zhao, Hongjun Gao, Xiya Ma, Xiaoqing Liu

**Affiliations:** ^1^Department of Pulmonary Oncology, The Fifth Medical Centre, Chinese PLA General Hospital, Beijing, China; ^2^Shanghai Tongshu Biotechnology Co., Ltd., Shanghai, China

**Keywords:** hepatoid adenocarcinoma of lung, α-fetoprotein, FAT atypical cadherin 1, genomic profiles, immunotherapy

## Abstract

Hepatoid adenocarcinoma of lung (HAL) is a rare and aggressive tumor. The current study reported a new HAL case in the right lower lung with high serum α-fetoprotein (AFP) level in a 71-year-old male patient. After the confirmation of morphology and immunohistochemistry, the patient was diagnosed clinically with HAL and treated with radio-frequency ablation. However, the patient whose disease progressed eventually died 4 months after diagnosis. Whole genome sequencing analysis identified a driver gene mutation in the FAT atypical cadherin 1 gene (*FAT1*) and the copy number loss. The tumor was microsatellite-stable and tumor mutation burden (TMB) was 1.69 mutations/Mb. PD-L1 expression was negative by IHC. Our finding provide further clues for the molecular basis of HAL and the efficacy of immunotherapy needs to be explored.

## Background

Hepatoid adenocarcinoma of lung (HAL) is a rare subtype of hepatoid adenocarcinoma (HAC) with poor prognosis. HAL was first formally described by Ishikura et al. (1) in 1990. It histologically resembles typical hepatocellular carcinoma (HCC) metastatic to the lung. At present, it is difficult to diagnose accurately and timely because the HAL patients rarely exhibit specific clinical manifestations. Therefore, the finding of HAL pathological features is important for the early diagnosis.

There are few effective treatment options in HAL patients. However, in recent years, immunotherapies have shown great promise in the treatment of cancer, in which, anti-programmed cell death 1 (PD1), and anti-PD1-ligand 1 (PD-L1) monoclonal antibodies have been approved by the Food and Drug Administration (FDA) for anticancer treatment, including non-small cell lung cancer (NSCLC). Clinically, the immunotherapeutic biomarkers are mainly PD-L1, microsatellite-instability (MSI), and tumor mutation burden (TMB). The PD-L1 expression is a logical biomarker for the prediction of response to anti-PD1/PD-L1 immunotherapies (2); tumors with MSI-high are exquisitely sensitive to PD-L1 inhibitor (3); and high TMB predicts a better response to immunotherapies (2). Besides, several effective therapies have been developed to target to specific genetic alterations in cancers. Therefore, we examined related biomarkers using whole genome sequencing (WGS) to evaluate new therapeutic possibilities in HAL. To the best of our knowledge, this is the first case of HAL using WGS to reveal some molecular clues and provide potential therapeutic options.

## Case Presentation

**Figure 1:**
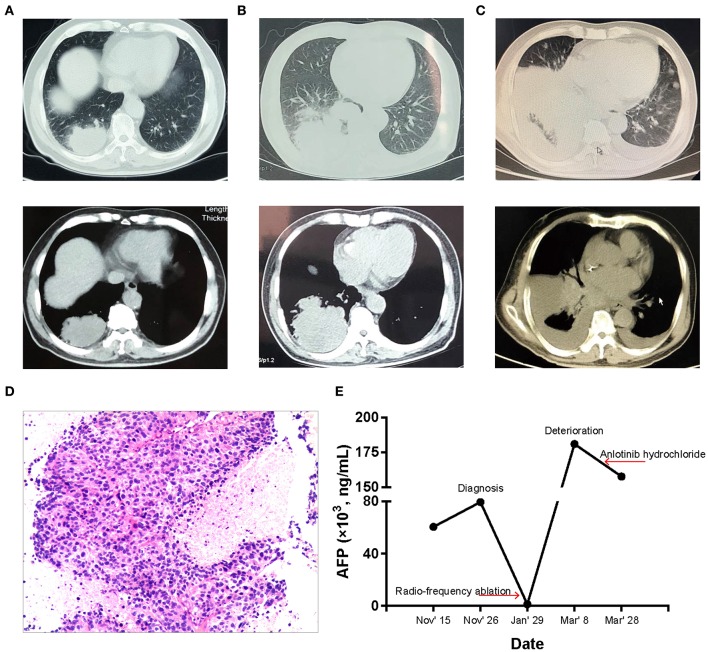
Patient condition during the clinical course. **(A)** CT images before treatment. There is a mass in the right lower lung with multiple nodules in the right lower lung. **(B)** CT images after radio-frequency ablation treatment. The mass in the right lower lung is larger than before. **(C)** CT images after exacerbation, which showed right lower lung cancer with bilateral pleural effusion, and contralateral new lesions. **(D)** Histological finding. Tumor cells showed adenoid and trabecular structures, which grew around the blood vessels. And the cell atypia and nuclear fission was obvious (hematoxylin and eosin staining; magnification, × 10). **(E)** AFP level during the clinical course.

The patient, a 71-year-old Chinese male non-smoker, had clinical history of lung space occupying lesion without any discomfort for 2 years. On October 26, 2018, he sought medical consultation for a 4-month history of stomachache, fatigue, and constipation at other hospital. The chest computed tomography (CT) showed a mass in the right lower lung, multiple nodules in the right lower lung, and multiple enlarged lymph nodes in the mediastinum (Figure 1A). Positron emission tomography/computed tomography (PET/CT) revealed a 7 × 4.5 cm necrotic mass in the right lower lobe of the lung and extended to the pleura. There were metastases to right hilar, mediastinal and right supraclavicular lymph nodes, and right ilium. The serum AFP level was 60,500 ng/ml and elevated to 79,480 ng/ml 10 days later. Subsequently, the patient underwent CT-guided percutaneous lung biopsy. Poorly differentiated cancer cells could be seen and showed adenoid and trabecular structures, which grew around the blood vessels. And the cell atypia and nuclear fission was obvious, resembling metastatic hepatocellular carcinoma (Figure 1D). Results of immunohistochemical (IHC) analysis were presented in Table 1. The Ki-67 score was observed to be 80%. Furthermore, the abdominal magnetic resonance imaging (MRI) examination showed no any hepatic or other digestive tumor. According to the findings above, the patient was diagnosed as HAL, and the clinical stage was IV (T4N3M1).

**Table 1:** Results of immunohistochemical staining and molecular profiling analysis on patient's tumor tissue.

**Immunohistochemistry**	
CK, SALL-4, CK18	Strongly positive
CK8, CK7, AFP, Hepatocyte, STAT-6, CD117	Focally positive
CK20, p63, p40, CK5/6, Syn, CD56, CGA, Vimentin, Calretinin, TTF-1, napsin-A, CD34, D2-40, ALK, PD-L1	Negative
**Molecular profiling**	
*EGFR, ALK, ROS1, PD-L1, BRAF, HER2, KRAS, MET, RET*	Wild type
*FAT1*	Mutated, Copy number loss
MSI	Stable
TMB	1.69 mutations/Mb

*CK, cytokeratin; SALL-4, spalt like transcription factor 4; AFP, α-fetoprotein; STAT-6, signal transducer and activator of transcription 6; Syn, synapsin; CGA, glycoprotein hormones, alpha polypeptide; TTF-1, thyroid transcription factor 1; ALK, anaplastic lymphoma kinase; PD-L1, programmed death 1 ligand; EGFR, epidermal growth factor receptor; BRAF, b-raf proto-oncogene; HER2, erb-b2 receptor tyrosine kinase 2; FAT1, FAT atypical cadherin 1; MSI, microsatellite instability; TMB, tumor mutation burden*.

The patient was first treated with radio-frequency ablation on January 10, 2019. CT showed larger tumor volumes compared to diagnosis (Figure 1B). While the serum AFP level had decreased to 1,210 ng/ml. In early February, the patient received a palliative radiotherapy for bone metastases. Then the patient was referred to our hospital for medical treatment owing to the deterioration of his condition on March 7, 2019. At that time, the patient also developed a type I respiratory failure (pH 7.493, PCO_2_ 33.5 mmHg, PO_2_ 60.9 mmHg) and hepatic function damage (ALT 56 U/l, AST 79 U/l), besides, the repeated CT scan (Figure 1C) showed double pneumonia, bilateral pleural effusion and minimal pericardial effusion. Serum AFP level was 180,909 ng/ml (Figure 1E). The above findings suggested that the patient did not have anti-tumor treatment indications.

**Figure 2:**
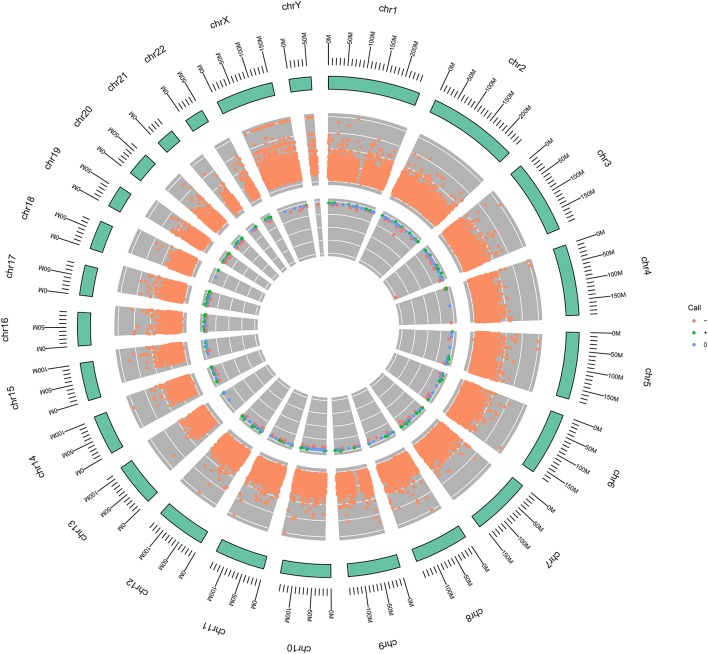
Circular genome diagrams of the patient. The outer circle (outside the green circle) represents the structure and scale of the chromosome. The middle circle indicates the somatic variation, in which, its Y-axis represents the allelic fraction (AF) value of each locus. 0 is the minimum and 1 is the maximum. The inner circle represents copy number variation (CNV). Orange color indicates deletion; green color indicates amplification, and blue color indicates neutral.

**Figure 3:**
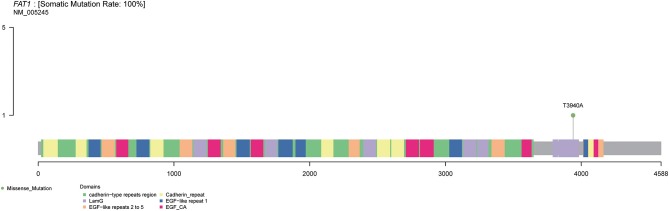
Schematic and simplified representation of FAT1 gene. Columns with different colors indicate different domains within the FAT1 gene, and the mutation site of FAT1 gene in the patient is indicated by the lollipop with green color.

Considering the negativity for PD-L1 expression by IHC and unresponsiveness to prior lines of therapies, WGS was performed to test related immunotherapeutic and genetic biomarkers, evaluating new therapeutic possibilities. Results showed that the tumor was microsatellite-stable and tumor mutation burden (TMB) was 1.69 mutations/megabase (Mb) (Table 1). The somatic variations and copy number variations (CNV) of the patient were showed in Figure 2. Further GO analysis was performed, and results suggested that these variations were not significantly located in any GO terms (adjusted *P*-value > 0.1) (Supplementary Figure 1). By referring to the driver genes list (4) most driver genes were detected to be wild type, except FAT atypical cadherin 1 (*FAT1*). The variant c.3940T>A in *FAT1* was found (Figure 3), as well as its copy number loss (Table 1). However, there are no targeted drugs or recommended therapies for the *FAT1* alternations at that time. The patient was given a series of palliative therapy, such as oxygen, anti-inflammatory and liver protection. Besides, the patient was treated with anlotinib hydrochloride, and his condition slightly improved with 2-weeks treatment. The serum AFP level had decreased. However, the patient's condition deteriorated and he died of respiratory failure on April 4, 2019.

## Discussion

Hepatoid adenocarcinoma (HAC) is a rare and aggressive tumor, in which, stomach is the most common primary site accounting for 63% while lung is one of the rarest originated organs accounting for only 5% (5). A review of 28 HAL cases found that most of the tumors occurred in men with a history of tobacco use, besides, a high serum AFP level was also noted (6). The patient we reported here did not have smoking history or any remarkable relevant family medical history. However, he developed HAL with an extremely high serum AFP level. Although most patients with HAL were detected to express AFP at a high level, there are exceptions (7, 8), leading to the proposal that AFP is not requisite for the diagnosis of HAL. Moreover, it was noteworthy that a patient with negative AFP expression had a 7-years survival time (9). Through a review of the literature, Papatsimpas et al. (10) suggested that the patients with normal AFP at presentation tend to have a longer overall survival time even after recurrence. Supportively, another case without AFP expression had a 9-years survival time (7). Here, the patient had an initial AFP level of 60,500 ng/ml, which might partially explain his short overall survival time.

Morphologically mimics HCC is the most uncontroversial feature of HAL. Lung is the most common organ for extrahepatic metastasis; thus, the exclusion of metastatic HCC is clinically relevant. The combination of morphology with immunohistochemical confirmation could be helpful in this regard. Haninger et al. studied and established an immunohistochemical panel to facilitate distinction (7). While in our cases, the staining results of IHC markers were not much in common with the findings of Haninger et al., which revealed an extremely heterogeneous feature of HAL immunohistochemistry. There still needs to integrate and analyze more HAL cases to find the immunohistochemical features, thus contributing to the accurate and timely diagnosis.

At present, the common treatments for HAL patients are surgical resection, chemotherapy and radiotherapy. Recently, Gavarancic et al. (11) reported a novel use of sorafenib in combination with platinum-based doublet chemotherapy in epidermal growth factor receptor (EGFR) wild-type HAL, which led to stable disease overall and achieved a survival among the longest reported for unresectable stage IV HAL. The patient in our report received a radiofrequency ablation treatment, which was a safe and effective treatment for the patients with advanced unresectable lung cancer (12). However, this treatment did not effectively stop the progress of HAL. Then, we performed genetic testing for making treatment decision. Unfortunately, neither actionable mutations nor biomarkers such as PD-L1, MSI was confirmed, indicating that it might be difficult for the patient to benefit from immunotherapy. The molecular analysis also revealed the wild-type status of genes commonly mutated in lung cancer, like *EGFR, ALK, ROS1, PD-L1, BRAF, HER2, KRAS, MET*, and *RET*. However, one driver gene mutation, *FAT1*, was detected. *FAT1*, one of the commonly mutated genes in lung adenocarcinoma, suppresses tumor growth through the activation of Hippo signaling, whereas promotes tumor migration via the induction of actin polymerization (13, 14). At present, there are no specific drugs targeted at *FAT1* alteration. While recently, Fang et al. demonstrated that *FAT1* mutation was associated with greater clinical response to anti-PD-L1 therapies in NSCLC, irrespective of TMB status (15). This indicates that the HAL patients with *FAT1* mutation may benefit from the anti-PD-L1 therapies. Furthermore, we also analyzed the genes with copy number variation in the Hippo signaling, and found that there was copy number loss on *FAT1*, as well as loss on *LATS1* (large tumor suppressor gene 1) and *NF2* (neurofibromin 2), suggested that deletion of tumor suppressor gene copy number might be associated with tumor development. Our findings were in line with the work of Morris et al. (16) that *FAT1* gene is deleted and mutated at a high prevalence across multiple human cancers, and its tumor suppressive properties.

In conclusion, HAL is a very rare cancer with very few cases reported, and trials with large amount of cases cannot be organized. This fact together with the extremely heterogeneous feature of HAL lead to the difficulty of accurate and timely diagnosis and treatment. And to our knowledge, this case represents the first report of HAL with *FAT1* mutation, which suggests some underlying mechanism of tumorigenesis. Further studies are needed to develop more effective treatment for HAL, especially immunotherapy.

### Bioinformatic Analysis

The sequencing data were firstly tested for quality control using fastqc. Next, the data were aligned to the human reference genome (NCBI build 37) using BWA (17), then sorted and removed PCR duplication using GATK 4.0 (18). Somatic mutation calling was performed using Mutect2 (18), VarDict (19), and Strelka2 (20). Somatic mutations existing in at least two of the results of the three software were selected as high confident mutations. The variant data were annotated using ANNOVAR (21), and converted to MAF files using maftools (22) for further analysis. The copy number variation was analyzed by GATK4.0 with default parameters following the tutorials of Broad Institute (Sensitively detect copy ratio alterations and allelic segments, https://software.broadinstitute.org/gatk/documentation/topic?name=tutorials). For the GO analysis, clusterProfiler R package (23) was used.

## Data Availability

The WGS data in this report can be found in the Sequence Read Archive (SRA, accession number PRJNA587384).

## Ethics Statement

The studies involving human participants were reviewed and approved by Ethics Committee of Chinese PLA General Hospital. The patients/participants provided their written informed consent to participate in this study.

## Author Contributions

JL, HQ, and BX contributed equally to the writing of the manuscript and designed the figures. JZ was involved in planning and supervised the study. HG interpreted the radiological images. XM performed the statistical analyses. XL reviewed and approved the final version of this work.

### Conflict of Interest

HQ and BX are employed by Shanghai Tongshu Biotechnology Co., Ltd. The remaining authors declare that the research was conducted in the absence of any commercial or financial relationships that could be construed as a potential conflict of interest.

## Supplementary Material

Supplementary Figure 1 Functional annotation. GO terms identified in the GO analysis for mutated genes in the categories biological pathways **(A)**, cellular component **(B)**, and molecular functions **(C)**. Each color indicates adjusted *P*-values.
