# Supplementary figures and images for: Genomic Profiles of a Patient of Pulmonary Hepatoid Adenocarcinoma With High AFP Level: A Case Report
Source: Front Oncol. 2019 Dec 11;9:1360. doi: 10.3389/fonc.2019.01360 (PMC6917606; doi:10.3389/fonc.2019.01360)

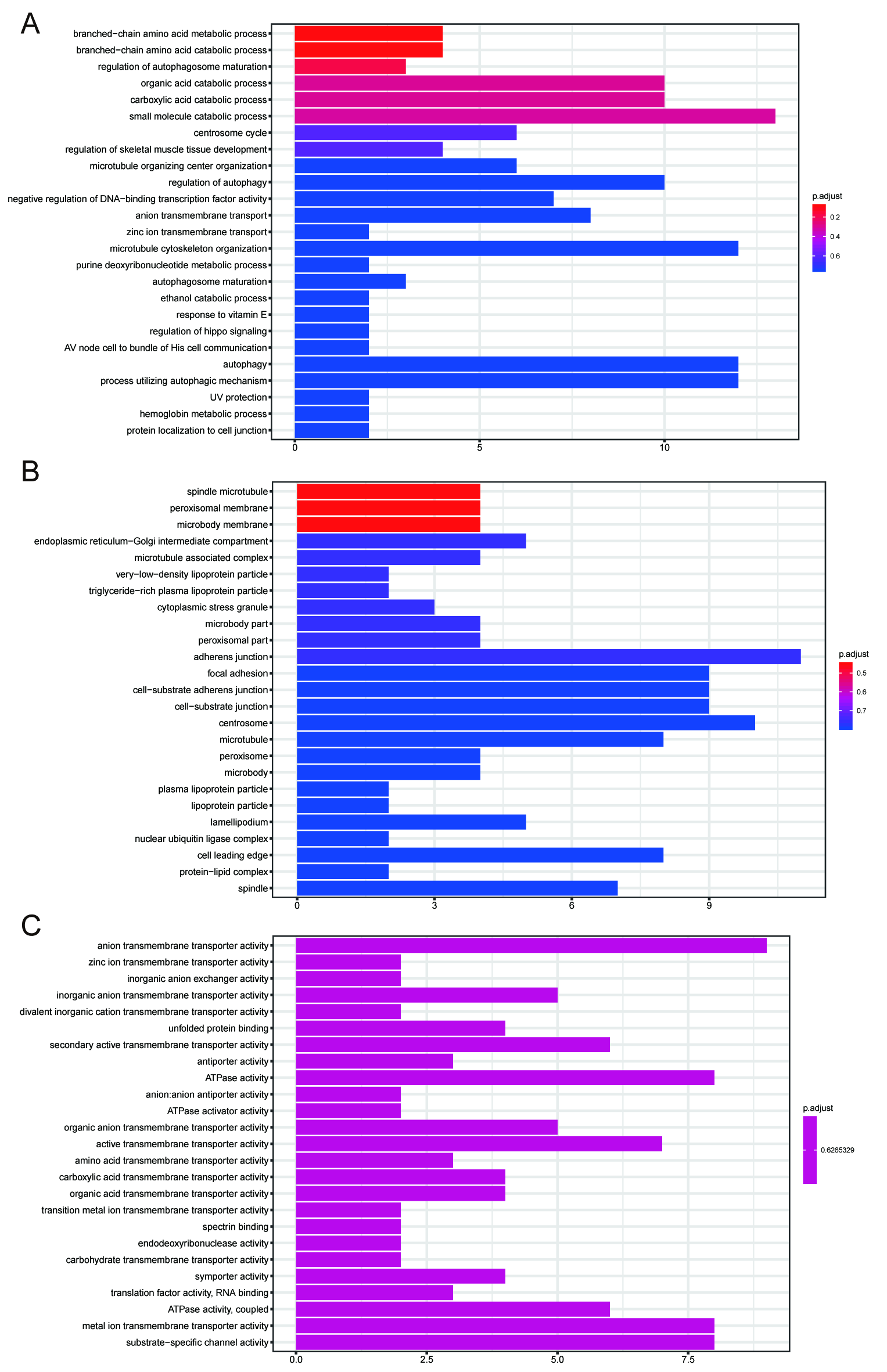

Supplement: Supplementary Figure 1 — Functional annotation. GO terms identified in the GO analysis for mutated genes in the categories biological pathways (A), cellular component (B), and molecular functions (C). Each color indicates adjusted P-values. [file Image_1.TIF]
